# Machine learning-based tool to assess risk of hemodynamically significant PDA in extremely premature infants

**DOI:** 10.1038/s41390-025-04489-w

**Published:** 2025-10-29

**Authors:** Erik Küng, Katharina Göral, Lukas Unterasinger, Christine Schilhart-Wallisch, Angelika Berger, Lukas Wisgrill, Georg Dorffner

**Affiliations:** 1 Division of Neonatology, Pediatric Intensive Care & Neuropediatrics, Department of Pediatrics and Adolescent Medicine, Comprehensive Center for Pediatrics, Medical University of Vienna, Vienna, Austria; 2 Institute of Clinical Biometrics, Center for Medical Data Science, Medical University of Vienna, Vienna, Austria; 3 Institute of Artificial Intelligence, Center for Medical Data Science, Medical University of Vienna, Vienna, Austria

## Abstract

**Background:**

A patent ductus arteriosus (PDA) is associated with complications in extremely preterm infants and its assessment requires trained personnel and equipment not always available. A prediction tool might guide the urgency and clinical decision making to allocate resources to patients with the highest risk. The aim of this study was to generate a clinical tool to assess the probability of a hemodynamically significant PDA in extremely preterm infants.

**Methods:**

An integrative review was performed, and potential risk factors were identified based on published research, pathophysiological hypotheses and clinical experience. Variables were selected based on stepwise regression with backward elimination and forward selection and then used to generate a nomogram for risk assessment in a single center retrospective study in 677 extremely premature infants.

**Results:**

A model comprising six variables was derived that achieved a sensitivity of 74.8% and a specificity of 53.4%, with an area under the ROC curve of 0.685 on an independent test set. This model was subsequently used for the creation of a nomogram.

**Conclusions:**

This is the first study to report a machine learning-based prediction tool for the risk assessment of hemodynamically significant patent ductus arteriosus using real-world data.

**Impact:**

- We report the first machine learning-based risk assessment tool for the prediction of a hemodynamically significant PDA.
- Several characteristics differ significantly between patients with spontaneous closure of the arterial duct and those with PDA, but a comprehensive and accurate assessment to predict hemodynamic significance is missing.
- The simple pen-and-paper score enables us to allocate risk for hsPDA using an easy-to-use pen-and-paper score with high accuracy. Risk assessment at an early stage might help guide targeted surveillance, prophylactic strategies, or individualized treatment decisions in extremely preterm infants.

## Introduction

The fetal ductus arteriosus is a large vessel that connects the main pulmonary trunk or proximal left pulmonary artery with the descending aorta and is essential in fetal circulation.^1,2^ It diverts cardiac output away from the lungs to the aorta to support systemic oxygenation. After delivery, post-natal circulatory adaptation depends on the closure of the ductus arteriosus.^2^ In healthy full-term neonates, functional closure of the ductus arteriosus physiologically occurs within 96 h after birth.^1^ Consequently, patent ductus arteriosus (PDA) refers to the failure of closure. In extremely low-birth-weight infants (birthweight <1000 g), spontaneous closure of the ductus arteriosus occurs in 26–34% of cases,^3,4^ in extremely preterm infants (born before 28 weeks’ gestation) in 24%,^5^ and in very low birthweight infants (birthweight <1500 g) in 69.4%.^6^ Persistent hemodynamically significant PDA arteriosus can be treated with early prophylaxis or after diagnosis with indomethacin,^1,7^ ibuprofen^1,7^ or paracetamol,^7^ as well as surgically with ligation^8^ or percutaneously,^9^ but whether or when it should be treated remains controversial.^1,2,10^ A left-to-right ductal shunt causes increased pulmonary blood flow and ductal steal from the systemic circulation, which might have adverse effects on premature infants, such as impaired oxygenation, increased need for respiratory support, or longer duration of mechanical ventilation, contributing to the development of bronchopulmonary dysplasia.^2^ In 2023, a multicenter randomized controlled study has shown that an expectant management for PDA is non-inferior to early ibuprofen treatment in infants born under 28 weeks of gestation with respect to necrotizing enterocolitis, bronchopulmonary dysplasia, or death at 36 weeks’ postmenstrual age.^10^ These results were confirmed in another multicenter randomized trial in 2024 in infants born under 28 weeks of gestation.^11^ However, a center specialized in the care for extremely preterm infants demonstrated that early hemodynamic screening led to a 23% absolute reduction in the combined outcome of death or severe BPD among infants born between 22 + 0 and 23 + 6 weeks gestational age, supporting a potential benefit of early identification and intervention in hemodynamically significant cases.^12^ The gold-standard for diagnosis is two-dimensional echocardiography combined with Doppler ultrasonography,^1,2^ which requires trained personnel and adequate equipment.

The possibility to predict which neonates are unlikely to spontaneously close their arterial duct could enable early identification of preterm infants at risk for hemodynamically significant PDA with the corresponding adverse effects as mentioned above, allowing for a personalized medicine approach. This would result in targeted management with the outlook of minimizing stress for the fragile infants, reducing unnecessary medication, and optimizing resource allocation.

The aim of this study was to generate a fast, reliable, and easily accessible tool to identify premature infants with a high-risk for hemodynamically significant PDA.

## Methods

### Patient cohort and ethical approval

**Fig. 1:**
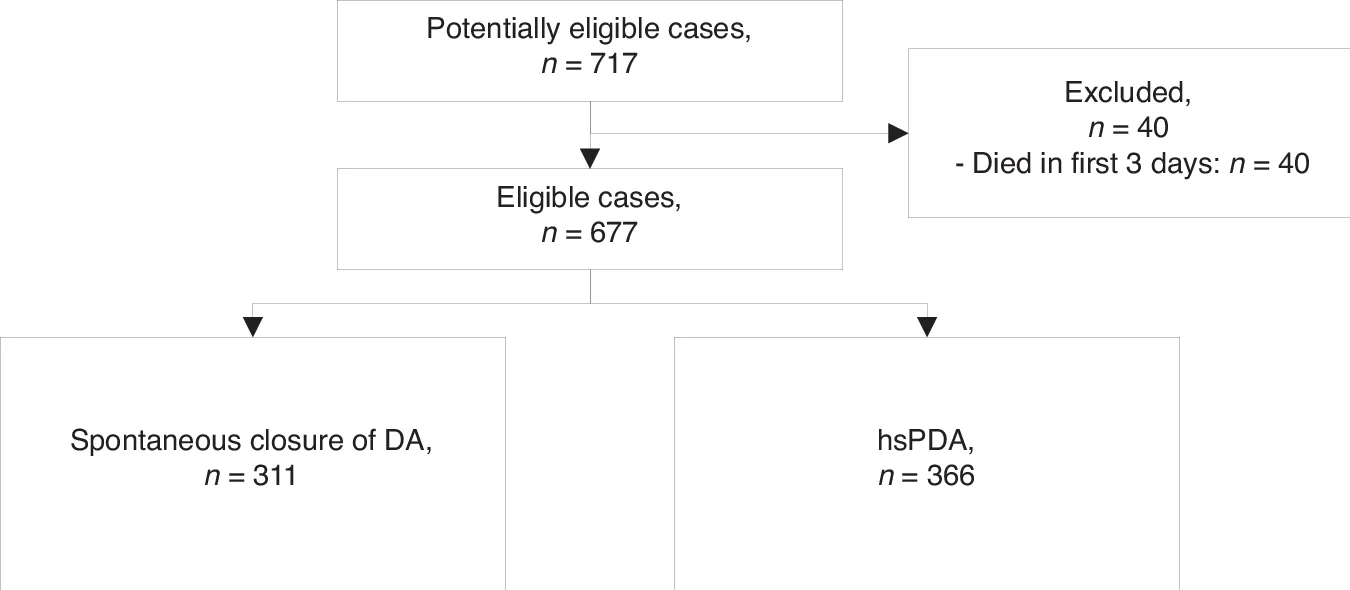
Patient flow chart for hsPDA cohort. Flow diagram depicting case screening, exclusions, and final allocation.

A retrospective single-center cohort study was performed following SAMPL^13^ and MINIMAR^14^ guidelines (Supplemental Table S1). The study was approved by the ethics committee of the Medical University Vienna (No. 2087/2019) and was conducted in accordance with the Declaration of Helsinki. All inborn neonates born <28 weeks’ gestation between November 2011 and August 2019 with data in our electronic patient data management system were included. Infants receiving surgery or who died in the first three days of life were excluded. The patient flow chart is depicted in Fig. 1, and patients characteristics are summarized in Table 1.

**Table 1:** Patient characteristics of the whole study population, the learning set, and the testing set and the percentages of the characteristics in this group (e.g., 14 patients (3%) of the Learning set had no antenatal steroids).

Parameters	All patients	Learning set	Testing set
Number of patients, *n* (%)	677	450 (66)	227 (34)
Female patients, *n* (%)	294 (43)	184 (41)	110 (48)
Gestational age, weeks	25.7 (1.4)	25.7 (1.4)	25.7 (1.4)
Birth weight, kg	0.79 (0.2)	0.79 (0.2)	0.78 (0.2)
Extremely low birth weight infants, *n* (%)	552 (83)	367 (83)	185 (83)
Maternal hypertension, *n* (%)	50 (7)	29 (6)	21 (9)
Antenatal steroids complete, *n* (%)	373 (55)	243 (54)	130 (57)
Antenatal steroids incomplete, *n* (%)	230 (34)	152 (34)	78 (34)
No antenatal steroids, *n* (%)	22 (3)	14 (3)	8 (4)
Vaginal delivery, *n* (%)	115 (17)	70 (16)	45 (20)
Delivery by cesarean section, *n* (%)	498 (74)	341 (76)	157 (69)
Delivery by emergency cesarean section, *n* (%)	62 (9)	37 (8)	25 (11)
Postnatal steroids in mg/kg in first 3 days	2.23 (3.45)	1.3 (2.7)	4.1 (4)
Surfactant mg/kg in first 3 days	252 (128)	257 (132)	242 (121)
Invasive ventilation, *n* (%)	253 (37)	160 (36)	93 (41)
Fluid intake in ml/kg in first 3 days	288 (112)	287 (104)	290 (127)
Minimal blood calcium level mmol/l in first 3 days	1.12 (0.12)	1.13 (0.12)	1.09 (0.12)
Mean FiO_2_ in % first 3 days	30 (13)	30 (14)	30 (12)
Maximum FiO_2_ in % in first 3 days	53 (29)	51 (29)	57 (29)
Maximum Interleukin-6 in pg/ml in first 3 days	486 (1640)	362 (920)	723 (2483)
Maximum C-reactive protein in mg/ml in first 3 days	1.59 (2.1)	1.65 (2)	1.47 (2.17)
Thrombocytes sum of three measurements in G/l in first 3 days	459 (186)	449 (183)	479 (191)
Gastric aspirates in ml/kg in first 3 days	11.1 (7.6)	12.4 (8)	8.6 (5.9)
Mean of mean arterial pressure in mmHg first 3 days	34.2 (4.6)	34 (4.7)	35 (4.4)
Standard deviation of mean arterial pressure in mmHg in first 3 days	5.5 (2)	5.4 (2)	5.5 (1.9)
Epinephrin in first 3 days, *n* (%)	15 (2)	9 (2)	6 (3)
Norepinephrin in first 3 days, *n* (%)	171 (25)	113 (25)	58 (26)
Mean arterial pH in first 3 days	7.27 (0.06)	7.26 (0.06)	7.28 (0.05)
PaO_2_ in mmHg in first 3 days	64.5 (12)	64.2 (12.6)	65.1 (10.7)
Mean heart rate in first 3 days	152 (10)	153 (11)	150 (9)
Standard deviation of heart rate in first 3 days	15 (5)	16 (6)	14 (4)
Percentage of time with SpO_2_ > 96%	23 (23)	22 (23)	23 (22)

### Definition of hsPDA

To address the difficulty of defining hsPDA, we used a negative definition. Non-hsPDA was defined as the absence of any pharmacological therapies used at our institution for PDA closure: indomethacin at any dosage, paracetamol >50 mg/kg/d for at least 3 consecutive days, or intravenous ibuprofen for at least 3 consecutive days. While paracetamol was also used for analgesia, this was restricted to a maximum of 30 mg/kg/day, justifying our chosen cut-off. At our institution, pharmacological therapy was always the first choice before ligation. Pharmacological therapy was initiated based on predefined targeted neonatal echocardiography criteria, including an enddiastolic flow in the left pulmonary artery (LPAd) of ≥0.2 m/s, and/or diastolic flow reversal in the abdominal aorta and/or in the celiac trunc, and/or a left ventricular load (visually estimated left ventricular ejection fraction, left atrium—LA/aorta—Ao >1.4, diameter of left ventricle in end diastol—LVEDD >15 mm/kg), provided a left-to-right shunt was present. Consequently, hsPDA was defined as non-hsPDA. Data were obtained from the electronic patient data management system (ICCA, Philips Healthcare, Amsterdam, Netherlands).

### Definition of risk factors associated with hsPDA

As a first step, an integrative review of the literature was performed to identify risk factors associated with the failure of spontaneous closure of the ductus arteriosus. In total, 17 variables were selected based on the available literature. In addition, we included 6 based on our hypothesis that these factors might be involved in the pathophysiology. A detailed summary and definition of the included variables is provided in Table 2.

**Table 2:** Variables and the method by which it was identified (integrative literature review (LR) or clinical experience (EXP)) with the respective definition, the *p* value when hsPDA (hemodynamically significant patent ductus arteriosus) is compared to no PDA, usage in the pen-and-paper tool (Model), and the reference.

Variable	Methods	Definition used	*p* value	Model	Literature
Birth weight	LR	Birth weight in kg	<0.001	yes	3, 4, 5–15
FiO_2_	LR	Mean FiO_2_ in first 3 days	<0.001	yes	5
		Maximum FiO_2_ in first 3 days	<0.001	no	
Bloodpressure	EXP	Mean blood pressure in first 3 days	<0.001	yes	-
		Standard deviation in first 3 days	<0.001	no	-
High SpO_2_	LR	% of time with SpO_2_ > 96% in first 3 days	<0.001	yes	22
Gestational age	LR	Gestational age at birth in weeks	<0.001	no	3, 5, 12–14
Volume expansion/high fluid intake	LR	ml/kg in first 3 days	<0.001	no	12,16
Increased arterial PaO_2_	EXP	Mean PaO _2_ in first 3 days	<0.001	no	23
Arterial pH	LR	Mean arterial pH in first 3 days	<0.001	no	14
Surfactant therapy	EXP	mg/kg in first 3 days	<0.001	yes	-
Heart rate	EXP	Mean heart rate in first 3 days	0.001	no	-
		Standard deviation of heart rate in first 3 days	0.001	no	-
Mode of delivery	LR	Vaginal delivery	0.001	yes	3
		Cesarean section	0.96	yes	-
		Emergency cesarean section	0.96	yes	-
Sepsis in the first 3 days of life	LR	Maximum interleukin-6 in first 3 days	0.005	no	4, 5, 17
		Maximum C-reactive protein in first 3 days	0.97	no	
Catecholamine support	EXP	Epinephrin yes or no	0.016	no	-
		Norepinephrine yes or no	0.99	no	-
Ventilation mode	EXP	Invasive ventilation in first 3 days	0.026	no	-
Antenatal steroids	LR	None	0.28	no	3, 18–20
		Antenatal steroids <24 h or >1week before birth,	0.49		
		Birth 24 h to 7 days after second antenatal steroid dose	0.25		
Platelets	EXP	Sum in first 3 days	0.45	no	17
Antenatal maternal hypertension	LR	Yes or no	0.47	no	3, 5
Hypocalcemia	LR	Minimal Ca level in first 3 days	0.56	no	12, 17
Sex	LR	Male or female	0.8	no	22
Postnatal steroids	LR	Hydrocortisone in mg/kg in first 3 days	0.91	no	21
Gastric aspirates	EXP	ml/kg in first 3 days	0.97	no	-
Small for gestational age	LR	Due to use of gestational age and birth weight, small for gestational age was not included	-	no	3

### Machine learning approach

Approximately two-thirds of the available data were used as a training set, selected based on the time of birth. The remaining one-third, consisting of cases that occurred after those in the training set, served as an independent test set.

In a first step, A ten-fold cross-validation on the training set was used to determine the superiority of one of the following models:

- A linear model, implemented as a perceptron (Neural network without hidden layer), using all available variables
- A nonlinear model, implemented as a multilayer perceptron (neural network with one hidden layer using 3 or 10 hidden units)
- A linear logistic regression model with forward feature selection
- A linear logistic regression model with backward feature elimination

For the neural networks, the Matlab neural network toolbox NETLAB from Aston University was used.^15^ Backward elimination and forward selection were performed using the respective procedures in IBM SPSS (Version 29) with default parameters. After selection of the best model, a nomogram for a pen-and-paper risk assessment was created using the rms package (https://github.com/harrelfe/rms)^16^ within the R environment (R version 4.3.3).^17^ Both models, as well as the nomogram version of one of the models, were then validated on the independent test set. To measure model performance, a ROC analysis was performed and the calibration of predicting PDA probabilities was illustrated in a calibration graph.

## Results

### Selection and definition of 22 risk variables for the PDA-model

Our review identified multiple clinical variables, Table 2, affecting the closure of the arterial duct: gestational age,^3,5,18–20^ birth weight,^3,4,18–21^ volume expansion,^18,22^ lower arterial pH,^20^ early onset-sepsis,^4,5,23^ and hypocalcemia^18,24^ are all associated with PDA. Cesarean section,^3^ being small for gestational age,^3^ antenatal maternal hypertension,^3,5^ antenatal steroids,^3,23,25,26^ postnatal steroids,^27^ and lower FiO_2_ levels^5^ were all associated with spontaneous closure of the arterial duct. Female infants were found to have a higher risk for hsPDA compared to male infants.^28^ The variables included based on pathophysiological hypothesis were: increased arterial PaO_2_^29^ and high oxygen saturation,^28^ platelet count,^23^ gastric aspirates, ventilation mode, and variations of continuously recorded vital parameters such as heart rate, blood pressure, and SpO_2_.

For constructing the model, the following variables were included in the pool of potential predictors. Aggregation was performed as minimum, maximum or sum and mean in the first 3 days of life. In our center, values are available every 15 min with routinely collected parameters (e.g., SpO_2_) or randomly (e.g., non invasive blood pressure): gestational age at birth (in weeks), birth weight (in kg), sex (male or female), antenatal steroids (none, incomplete, complete), mode of delivery (vaginal delivery, cesarean section, emergency cesarean section), surfactant (mg/kg in the first 3 days), ventilation (non-invasive or invasive), fluid intake (ml/kg in first 3 days), blood calcium levels (minimum in first 3 days), FiO_2_ (mean FiO_2_ in first 3 days and maximum FiO_2_ in first 3 days), interleukin-6 (maximum in first 3 days) and C-reactive protein (maximum in first 3 days), % of time with SpO_2_ > 96%, platelets (sum of three measurements in first 3 days), gastric aspirates (ml/kg in first 3 days), blood pressure (mean in first 3 days and standard deviation in first 3 days), epinephrine (yes or no), noradrenalin (yes or no) heart rate (mean in first 3 days and standard deviation in first 3 days), arterial pH (mean in first 3 days). The used parameters are summarized in Table 2.

### Characteristics of the patient cohort

Of 717 included patients, 677 (94.4%) survived the first 3 days and were used for statistical analyses; 40 (5.6%) died in the first 3 days and were excluded. 366 (54%) patients had no hsPDA, while 311 (46%) received therapy and therefore had a hsPDA. The mean gestational age was 25 + 5 weeks gestation with a standard deviation of 10 days, the mean birthweight was 0.79 (0.2) kg, and there were 294 females (43%). Further patient characteristics are shown in Table 1, including the variables with the corresponding mean (standard deviation) or number (percent) in the training and test set.

### Variable selection

31 cases (4.5%) had missing values in at least one variable. These were imputed by the mean value of the respective variable for the other cases. A total of 450 cases (all temporally preceding the remaining 227 cases in the test set) were selected as the training set.

Linear and nonlinear models are compared in Table 3 based on ten-fold cross-validation in the training set.

**Table 3:** Mean areas under the ROC curve from a ten-fold-cross-validation using either a linear model (perceptron) or multilayer perceptrons (MLPs) with 1 and 10 hidden units, respectively, on all features.

	AUC		
	Mean	± Standard deviation	*P* (t-test)
Linear perceptron	0.662	0.058	
MLP with 3 HU	0.604	0.077	0.0189
MLP with 10 HU	0.541	0.104	<0.001
Forward selection	0.638		0.080
Backward elimination	0.645	0.077	0.270

*P* values refer to a t-test comparing the performance with that of the linear perceptron.

Results show no superiority of nonlinear models, which is consistent with previous observations in the literature (11). Both backward elimination and forward selection led to a slight decrease in average performance. Since this decrease was not statistically significant, the model with the smallest number of variables on average was chosen as the final model, namely the one created by backward selection. This model comprised 6 variables and achieved a sensitivity of 74.8% and a specificity of 53.4% at a probability threshold of 0.5, with an area under the ROC curve of 0.685 in an independent test set.

The model achieved an area under the ROC curve of 0.720 for 2, 0.750 for 3, and 0.823 for >3 courses of therapy with either paracetamol, ibuprofen, or indomethacin.

**Fig. 2:**
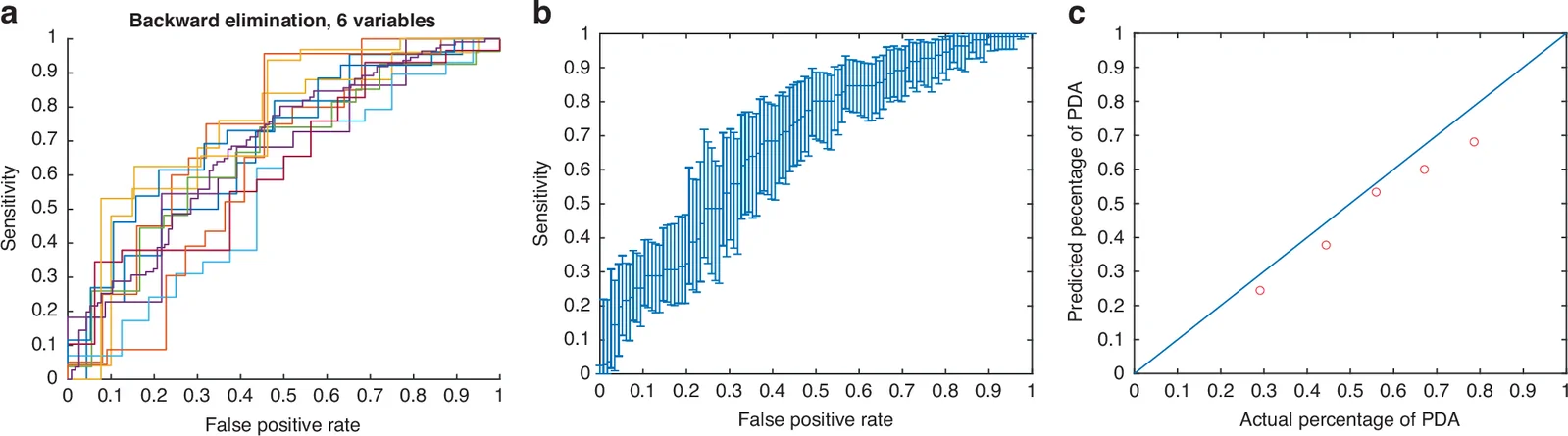
Discrimination and calibration of the predictive model. **a** ROC curves based on a ten-fold cross-validation in the training set with a mean AUC of 0.675. **b** ROC curves of selected best model on independent test set of 227 cases, including confidence bounds. **c** Calibration graph of the selected best model on the independent test set with the predicted PDA probability vs. actual relative frequency of PDA.

**Fig. 3:**
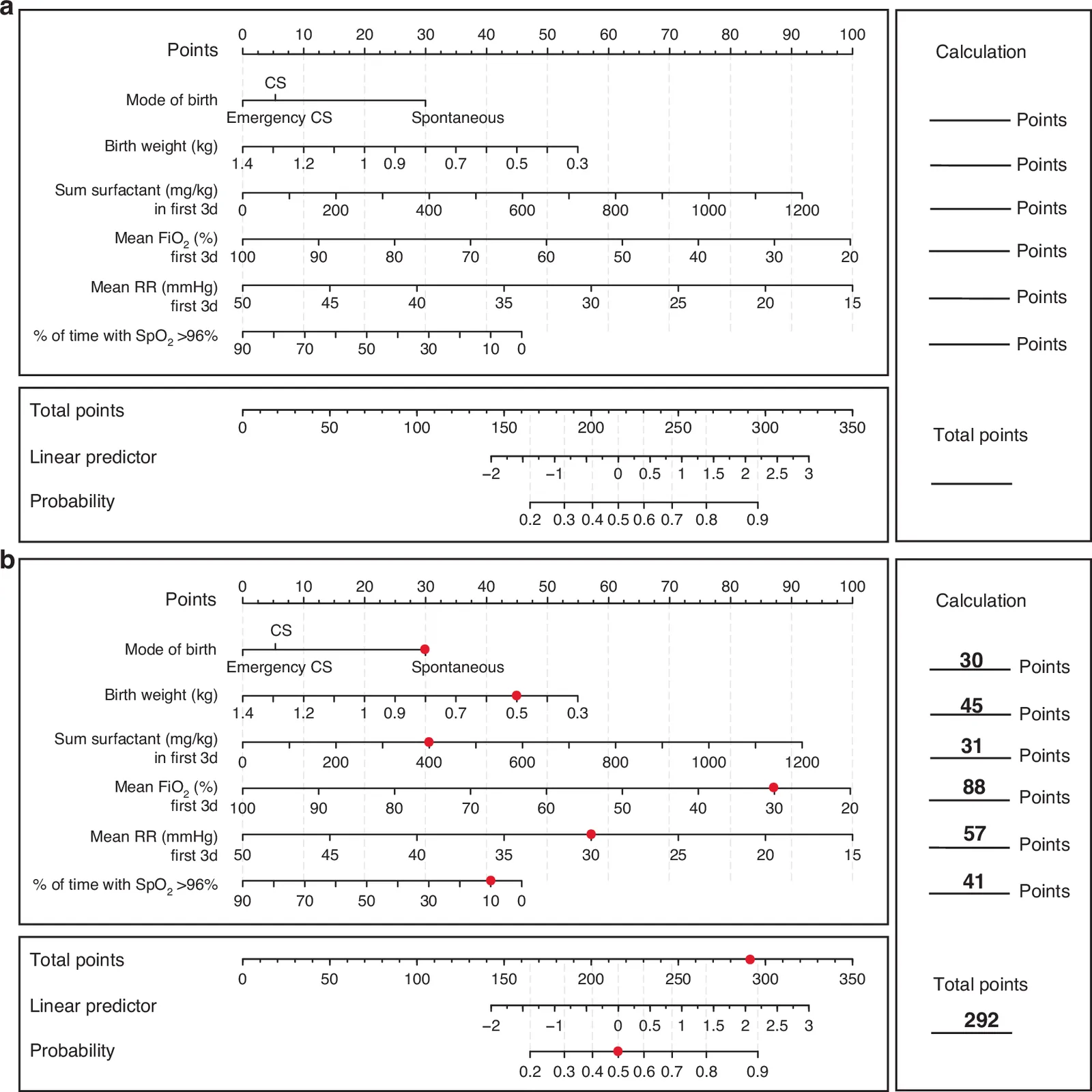
Nomogram for individualized prediction of hsPDA. **a** Nomogram to determine risk for PDA with a clinical example depicted in **b** a preterm infant born spontaneously (30 points) with a birth weight of 0.5 kg (45 points) who received 400 mg/kg surfactant in the first 3 days (31 points), had a mean FiO_2_ of 30% (88 points), a mean of mean arterial pressure of 30 mmHg (57 points), and a percentage of time with SpO_2_ > 96% of 10% (41 points) results in 30 + 45 + 31 + 88 + 57 + 41 = 292 points and therefore a probability of 88% for hsPDA.

Figure 2 displays the ROC curve with confidence intervals and the calibration of the final model in the test set, which can be considered reasonable. A nomogram was created based on that final model and the selected variables were mode of birth, first body weight, average blood pressure in the first 3 days, surfactant per kg over the first 3 days, average FiO_2_ in the first 3 days and percent of time with SpO_2_ > 96% in the first 3 days. The nomogram is depicted in Fig. 3 and should be applied after the first 3 days of life. This is a clinical example how the nomogram is applied: a preterm infant born spontaneously (30 points) with a birth weight of 0.5 kg (45 points) who received 400 mg/kg surfactant in the first 3 days (30 points), had a mean FiO_2_ of 30% (88 points), a mean of mean arterial pressure of 30 mmHg (57 points), and a percentage of time with SpO_2_ > 96% of 0% (45 points) results in 30 + 45 + 30 + 88 + 57 + 45 = 295 points and therefore a probability of 90% for hsPDA.

## Discussion

This is the first study to generate a nomogram-based risk assessment tool for the development of a hsPDA in extremely premature infants. Using linear regression models with backward elimination and forward selection of variables, a model with six variables was derived that achieved a sensitivity of 74.8% and a specificity of 53.4%, with an area under the ROC curve of 0.685 on an independent test set. These results are similar to Furzan et al.^18^ who used a combination of obstetric estimate of gestational age, race, mean fluid volume intake in the initial 24 h, and early treatment with volume expanders at 24 h after birth in very low birthweight infants to correctly classify 73% infants with hemodynamically significant PDA (79% with hsPDA and 79% without PDA).^18^ This is consistent with our AUC of 0.685 but different from our detection of 74.8% of PDA cases (83 out of 111) and of 53.4% of non-PDA cases (62 out of 116) in an independent test set.

Although not designed for it, we tested the model for its AUC for multiple therapies and found increasing AUCs up to 0.823 for >3 courses of therapy with either paracetamol, ibuprofen, or indomethacin.

Overall, the variables selected by the integrative review were significantly different between infants with hsPDA and infants without, with some interesting exceptions. Female sex was not significantly different between the two groups in our dataset, which is consistent with a recent systematic review and meta-analysis.^30^ Furthermore, minimal blood calcium levels, platelet count, and gastric aspirates were not significantly different. Although the association was hypothesized to be pathophysiologically plausible, it could not be validated with this method in our dataset. Interestingly, maximum C-reactive protein was not significantly different while maximum interleukin-6 levels were. This might be due to the 24-h lag of C-reactive protein behind interleukin-6.^31^ Additionally, elevated interleukin-6 values might solely reflect perinatal inflammation rather than a microbial-related infection.

The variables selected based on pathophysiological hypothesis were increased arterial PaO_2_^27^ and high oxygen saturation^26^ because they might influence the DA, and platelet count may be crucial for DA closure by promoting thrombotic sealing and luminal remodeling of the constricted duct.^21^ Gastric aspirates and subsequently protracted feeding might be an indirect sign of hsPDA. Lastly, ventilation mode and variations of continuously recorded vital parameters such as heart rate, blood pressure, and SpO_2_ might be indicative for hsPDA.

The variables selected for nomogram creation were mode of birth, first body weight, surfactant per kg in the first 3 days, mean FiO_2_ in the first 3 days, mean of mean arterial pressure in the first 3 days, percent of time >96% SpO_2_, and average blood pressure in the first 3 days. While mode of birth,^3^ birthweight,^3,18–21,30^ mean FiO_2_,^5^ high SpO_2_,^28^ and low blood pressure^32^ are known factors associated with hsPDA. In addition, FiO_2_ and SpO_2_ might be influenced by mean airway pressure, but this interaction and influences is not studied yet.

Interestingly, the percentage of non-hsPDA of 54% in our cohort of extremely preterm infants is rather high when compared to the rate of spontaneous closure in the literature. In extremely low birth weight infants, spontaneous closure rates of 34^3^ and 26%^4^ have been reported, whereas in very low birth weight infants, rates as high as 69.4% have been observed.^6^ In our study, we could not differentiate between DA closure and non-hsPDA, this might account for the difference, furthermore all of those studies were performed before 2010, and the management has changed since then, which might contribute to the fact that our reported rate is closer to the rate reported in very low birth weight infants, although our patients are infants born before 28 weeks’ gestation. Our management of extremely preterm infants consisted of a 3-day resting phase with minimal handling, including no PDA assessment or PDA therapy.

This study is limited by its retrospective design, including only one study center. Furthermore, the study included neonates born between 2011 and 2019. The clinical management changed in these 8 years, and the number of infants with lower gestational age increased. Although this was taken into account when selecting the training and test data sets, there is a potential bias. Only external validation can show and clear the model from this potential bias inherent to single center retrospective studies.

In addition, our definition of hsPDA was based on therapy using echocardiography for indication, with each echo criteria having uncertainties for itself, and potentially other clinical parameters influencing the decision of the clinician in charge. We believe that the first step in developing a new tool for risk assessment is a literature review, retrospective study, a model calculation and then peer review publication, allowing prospective validation and improvement of said model. The next step could be a reproduction of this study in a comparable center, a prospective observational study in the same and especially in a different center to validate our results and subsequently using the new prospectively collected data to retrain and hopefully improve the model.

Our model results are comparable to previous studies. Additionally, the clinical parameters we obtained were recorded using an electronic patient file system with high temporal (first hour every 5 min, and then every 15 min) resolution. This approach ensured the acquisition of reliable and continuous data, facilitating the calculation of mean values with enhanced accuracy and validity. Such precise data collection would be challenging to achieve with paper-based medical documentation systems. To allow for comparison and implementation, we used the mean as easy to apply aggregation with considerable robustness.

In conclusion, this study is the first to present an easy-to-use pen-and-paper tool for assessing the probability of hemodynamically significant ductus arteriosus. This assessment tool might allow for better resource allocation, targeted diagnostics, or targeted therapy, as well as preventing overtreatment. Our scoring system can be easily used as a pen-and-paper tool, but demonstrates potential for seamless integration into electronic patient file systems, enhancing its utility and applicability in clinical practice. Caution should be exercised to avoid missing critical congenital heart disease, as DA patency may be essential for maintaining systemic or pulmonary blood flow. Further studies are needed and planned to validate and further optimize the PDAR tool for clinical application.

## Supplementary information


Supplementary Tables


## Data Availability

The datasets analyzed during the current study are available from the corresponding author on reasonable request.
