# Supplementary material for: Machine learning-based tool to assess risk of hemodynamically significant PDA in extremely premature infants
Source: Pediatr Res. 2025 Oct 29;100(1):270–6. doi: 10.1038/s41390-025-04489-w (PMC13524808; doi:10.1038/s41390-025-04489-w)
Supplement: Supplementary file 1 [file 41390_2025_4489_MOESM1_ESM.pdf]

Supplementary Table S1: MINIMAR table

Study population and setting	
Population	Inborn neonates born < 28 weeks' gestation
Study setting	European academic perinatal centre
Data source	Patient data management system
Cohort selection	Exclusion of infants who died or needed surger in the first 3 days of life.
Patient demographic characteristics	
Age	Mean gestational age in weeks 25.7 (1.4)
Sex	Female 43 %
Race	Not provided
Ethnicity	Not provided
Socioeconomic status	Not provided
Model architecture	
Model output	hsPDA
Target user	Clinician taking care of preterm infants
Data splitting	2/3 as training set, 10-fold cross valudation; 1/3 as test set
Gold standard	Patients with treatment for PDA defined as hsPDA
Model task	Prediction
Model architecture	A linear logistic regression model with backward feature elimination
Features	Provided in detail in table 2
Missingness	Missing data were imputed by the mean value of the respective variable for the other cases.
Model evaluation	
Optimization	Not performed
Internal model validation	Intern 10-fold cross-validation
External model validation	Not performed
Transparency	Code available on request
